# The gut microbiota–brain axis in neurological disorder

**DOI:** 10.3389/fnins.2023.1225875

**Published:** 2023-08-04

**Authors:** Hanif Ullah, Safia Arbab, Yali Tian, Chang-qing Liu, Yuwen Chen, Li Qijie, Muhammad Inayat Ullah Khan, Inam Ul Hassan, Ka Li

**Affiliations:** ^1^Department of Nursing, West China Hospital, West China School of Nursing, Sichuan University, Chengdu, China; ^2^Key Laboratory of Veterinary Pharmaceutical Development, Ministry of Agriculture, Lanzhou, China; ^3^Key Laboratory of New Animal Drug Project of Gansu Province, Lanzhou, China; ^4^Lanzhou Institute of Husbandry and Pharmaceutical Sciences, Chinese Academy of Agricultural Sciences, Lanzhou, China; ^5^State Key Laboratory of Biogeology and Environmental Geology, China University of Geosciences, Wuhan, China; ^6^Department of Microbiology, Hazara University Mansehra, Mansehra, Pakistan

**Keywords:** microbiota, neurological disorders, gut-brain axis, signaling pathways, gut dysbiosis

## Abstract

The gut microbiota (GM) plays an important role in the physiology and pathology of the host. Microbiota communicate with different organs of the organism by synthesizing hormones and regulating body activity. The interaction of the central nervous system (CNS) and gut signaling pathways includes chemical, neural immune and endocrine routes. Alteration or dysbiosis in the gut microbiota leads to different gastrointestinal tract disorders that ultimately impact host physiology because of the abnormal microbial metabolites that stimulate and trigger different physiologic reactions in the host body. Intestinal dysbiosis leads to a change in the bidirectional relationship between the CNS and GM, which is linked to the pathogenesis of neurodevelopmental and neurological disorders. Increasing preclinical and clinical studies/evidence indicate that gut microbes are a possible susceptibility factor for the progression of neurological disorders, including Alzheimer’s disease (AD), Parkinson’s disease (PD), multiple sclerosis (MS) and autism spectrum disorder (ASD). In this review, we discuss the crucial connection between the gut microbiota and the central nervous system, the signaling pathways of multiple biological systems and the contribution of gut microbiota-related neurological disorders.

## 1. Introduction

Human health is seriously threatened by the dramatic environmental and lifestyle changes of the modern era. An unprecedented rise in a diverse range of neurological disorders is one of the major global challenges. Since the last decade, it has been evident that the gut microbiota has a potential role in brain function by mediating signaling pathways through microbial metabolites (Grochowska et al., 2019; Iannone et al., 2019). At the connection of neuroscience and microbiology, groundbreaking studies, largely conducted over the past ten years, have revealed active relations between animals and the microbial populations that live inside them that support the development and operation of neurological systems. These interactions, which involve immunological, neural, and chemical communication, are complex, but they are vital to the health of individuals and our understanding of neurological disorders (Morais et al., 2021). The gut microbiota residing in the gastrointestinal (GI) tract plays an important role in the health status of the host by regulating cells in local and distant organs, including the brain. Bidirectional transmission occurs in the gut–brain axis (GBA) in the form of a two-way communication mechanism between the gut and the neurological system of the host. This information can be transferred through brain networks, hormones, and the immune system, which facilitate the intestinal microbiota. Bidirectional transmission in the GBA regulates brain dysfunction mechanistically, maintains a mutualistic association with the host and regulates the innate and adaptive immune systems (Collins et al., 2012; Carabotti et al., 2015). This axis involves different pathways, such as the autonomic and enteric nervous system, the endocrine system, the hypothalamic–pituitary–adrenal axis (HPA), the immune system, and the microbiota and its metabolites (Blaser, 2017; Burberry et al., 2020). A healthy gut microbiota benefits the host by producing microbial metabolites and neurotransmitters for communication with host cells, such as intestinal epithelial cells (IECs) and immune cells. Alterations in the gut microbiota and microbial metabolite production have been linked to a wide range of immune-related neurological disorders, including developmental disorders, neurodegeneration, and emotional dysregulation. The brain is the organ responsible for all of an individual’s behavior and for controlling it. It is composed of many diverse populations of neuronal and nonneuronal cells that are connected by incredibly sophisticated structural networks (Deidda and Biazzo, 2021). The digestive tract (GI) is the habitat for more than 98% of the bacteria in our bodies. The term “gut microbiota” refers to the particular microorganisms that are present and reside in the gut (Ma et al., 2019).

The development of omics techniques has contributed to the understanding of the gut microbiota as one of the key regulators of the interactions between the gut and the brain (Bhattarai et al., 2021; Zhu et al., 2022). Animal and human research has provided evidence that the gut microbiota might influence brain behavior and cognitive development by producing hormones, immunological factors, and metabolites, which also suggests that changing the gut microbiome may improve or potentially treat brain disorders (Lee et al., 2011; Braniste et al., 2014; Jasarevic et al., 2015; Ogbonnaya et al., 2015; Yano et al., 2015; Wang and Wang, 2016). Signals from the brain can affect the sensorimotor and secretory functions of the stomach through intricate neurohumoral networks, and likewise, visceral afferent signals coming in the gastrointestinal tract can affect brain function (Cryan and Dinan, 2012). The gut-brain axis has recently emerged as a key participant in the regulation of normal brain functioning under physiologically normal conditions as well as in the development of neuropathological diseases as a risk factor or condition (Ma et al., 2019).

However, there is a lack of widespread confirmation of the mechanisms underlying links between the gut microbiota and brain disorders (Martin et al., 2018). New technologies are being created to go beyond correlative research and find and validate biological mechanisms of action that have the real potential to treat human disease. In this review, we discuss the interaction between the gut and brain and their signaling pathways. Furthermore, we discuss the function of the microbiota and neurological disorders such as neuropsychiatric disorders (schizophrenia and ADS), mood disorders (anxiety and depression), and neurodegenerative disorders (PD, AD, and MS).

## 2. Gut microbiota-brain axis

The gut microbiome consists of bacteria, archaea, viruses, and eukaryotic microbes that colonize the digestive tract. The gut microbiota, which comprises approximately 100–150 times more genes than the human genome, is found in the human intestines and includes approximately 1,000 species and 7,000 types of bacteria, gram-positive or gram-negative Firmicutes (including the species *Lactobacillus, Eubacterium,* and *Clostridium*), and gram-negative Bacteroidetes form the majority of the bacteria (containing *Bacteroides* and *Prevotella*) (Flowers and Ellingrod, 2015; Blaser, 2017; Askarova et al., 2020; Tarawneh and Penhos, 2022). The following five phyla make up the majority of the gut microbial community: Bacteroidetes, Firmicutes, Actinobacteria, Proteobacteria, and Verrucomicrobia (The Human Microbiome Project Consortium, 2012). Individuals’ diet, age, gender, environment, and genes had an impact on the composition of their gut microbiota (Takagi et al., 2019). Dysbiosis of the human gut microbiome has been associated with various pathologies (Perry et al., 2016). Gut dysbiosis, as shown by variations in the diversity and frequency of the microbial community (overall taxa and species) that comprise the gut flora, has been connected in both animal and human studies to abnormal brain protein aggregation, inflammation, immune dysregulation, and reduced neuronal and synaptic activity studies of AD (Cryan et al., 2020; Gubert et al., 2020).

**Figure 1:**
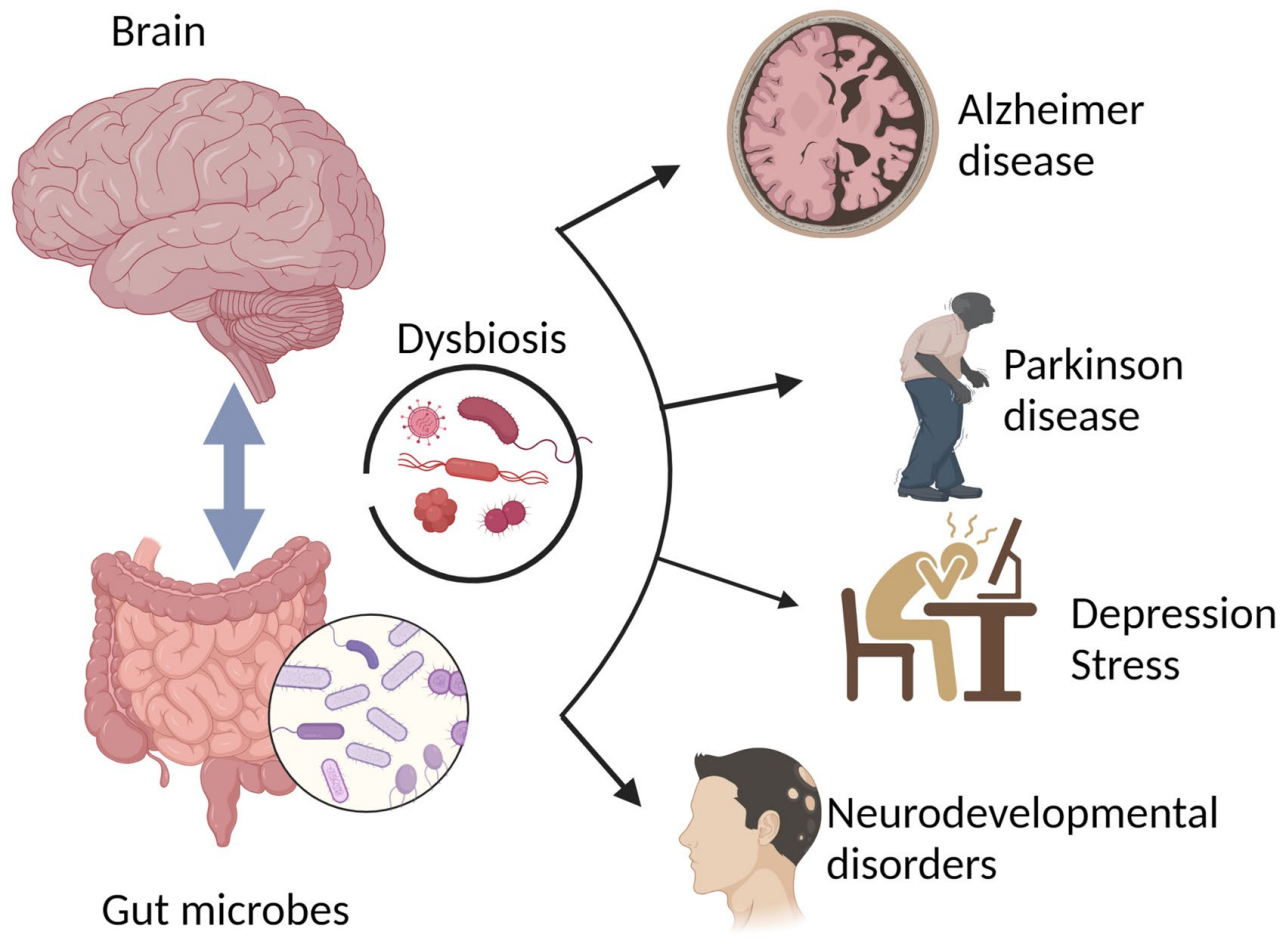
The physiological homeostasis attained during typical brain functioning is a result of the interactions between the brain and the gut-brain (gut microbiota). Several brain disorders, including Parkinson’s disease, neurodegenerative diseases, depression, stress, Alzheimer’s disease, and neurodevelopmental disorders, have been linked to altered gut microbiota or gut dysbiosis.

The capability of the gut microbiota to affect brain-related activities suggests that it triggers the production of immune factors that target both the CNS and the enteric nervous system (ENS), such as cytokines and inflammatory mediators (Wood and Galligan, 2004). The autonomic nervous system, a component of the peripheral nervous system, regulates physiological processes not subject to conscious control. It controls vital visceral functions by coordinating complimentary responses between the sympathetic and parasympathetic nervous systems. Understanding the bidirectional communication between the CNS and the digestive tract was greatly advanced by the discovery of the ENS, a branch of the autonomic nervous system. The ENS, sometimes known as the “second brain in the body,” is maintained in a healthy state by the cooperation of enteric neurons and connections to the CNS (Rao and Gershon, 2018). The ENS is made up of millions of neurons that are found in the mucosa of the digestive tract. These neurons are responsible for maintaining the equilibrium of intestinal activities. The most direct route of communication between the gut and the brain is the vagus nerve (de la Fuente-Nunez et al., 2018). A deeper understanding of the gut-brain connection showed a complex communication pathway that not only maintains the health of the gastrointestinal system but is also likely to have a variety of consequences on how the brain functions as a whole, including higher cognitive function and motivation (Rhee et al., 2009). The gut-brain axis (GBA), which is a sophisticated bidirectional communication network between the intestine and the CNS, is where communication occurs between the CNS and intestine (Figure 1; Sudo et al., 2004; Rao and Gershon, 2018; Skonieczna Zydecka et al., 2018). The routes of communication involve the autonomic nervous system [for example, the enteric nervous system (ENS) and the vagus nerve], the neuroendocrine system, the hypothalamic–pituitary–adrenal (HPA) axis, the immune system and metabolic pathways (Duvallet et al., 2017; Blacher et al., 2019; Burberry et al., 2020). Several neurotransmitters (Yano et al., 2015; O'Keefe, 2016) and metabolites, including short-chain fatty acids, secondary bile acids, vital vitamins, and amino acids (Ellwardt et al., 2016; Engelhardt et al., 2016; Mittal et al., 2017), modulate many immune system pathways (Baj et al., 2019; Dalile et al., 2019), which in turn affect cognition, behavior, learning, movement, and neurodegenerative diseases (Jenkins et al., 2016; Kennedy et al., 2017; Feng et al., 2020). The gut-brain axis has been termed the GMB axis since it appears to regulate the immune system, digestive tract, behavior, stress response, and CNS processes (Savignac et al., 2011; Collins et al., 2012; De Palma et al., 2014; Fond et al., 2015; Pirbaglou et al., 2016; Rincel and Darnaudery, 2020). Notably, advancements in gut microbiota sequencing have revealed a strong relationship between the complex ecosystem and the CNS (Knight et al., 2018). In recent years, there has been increasing interest in studying interactions between the brain, gastrointestinal microbiome and their bidirectional relationship.

## 3. How the gut microbiota affects the brain

**Figure 2:**
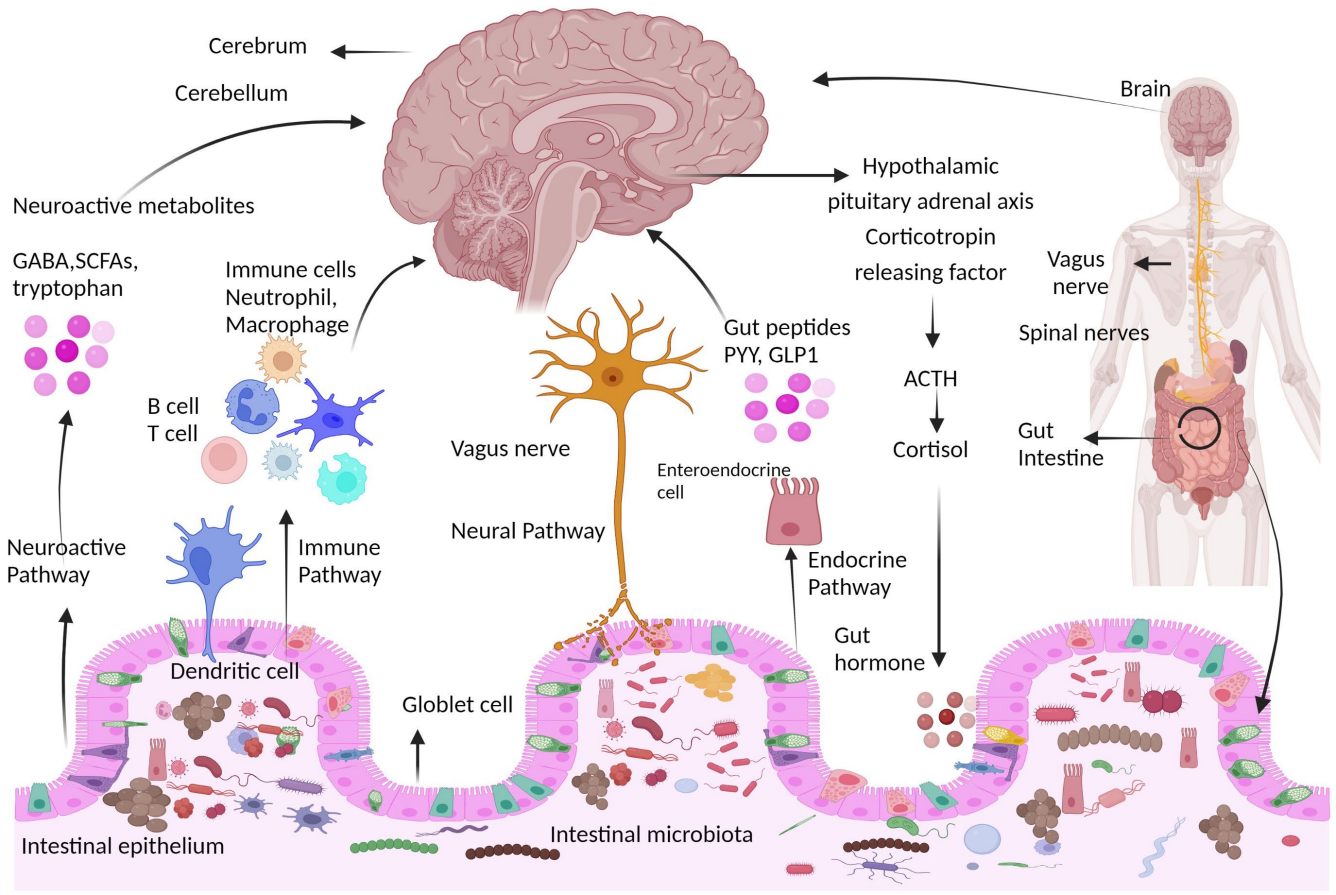
Communication pathways between the brain and gut microbiota. The interaction between the central nervous system (CNS) and gut microorganisms is mediated via several direct and indirect gut-brain axis mechanisms. They include the immune pathway (including cytokines), short-chain fatty acids and microbial metabolites; the neuroactive pathway, including neurotransmitters and neuroactive metabolites; the neural pathway [enteric nervous system, vagus nerve, and spinal nerves (Sgritta et al., 2019); and the endocrine pathway, hypothalamic pituitary adrenal axis (HPA) (Lyte, 2014b)]. HPA axis response that involves neurons of the hypothalamus that release hormones such as corticotropin receptor hormone (CRH) into the portal circulation or the brain, causing the release of the hormone adrenocorticotropic hormone (ACTH), which starts the production of cortisol and its release. The neuroimmune signaling reactions are regulated by cortisol.

The CNS and ENS communicate with one another using a number of chemical signaling mechanisms, including direct neuronal, immune, and endocrine pathways (Yoo and Mazmanian, 2017). The gut-brain axis is a network of connections involving multiple biological systems that facilitates bidirectional communication between gut bacteria and the brain and is vital for maintaining the gastrointestinal, neurological, and microbial systems of animals (Martin et al., 2018; Cryan et al., 2019). In addition to the neurological system, the gut microbiota also affects the brain through the endocrine, immunological, and metabolic systems (the gut-brain neuroanatomical pathway) (Cryan and Dinan, 2012; Montiel-Castro et al., 2013). In the gut microbiota-brain axis, more emphasis is placed on the involvement of bacteria because the gut microbiota can be used as an independent variable and modified intentionally (Al Omran and Aziz, 2014). Microbes can affect how the nervous system develops, matures, ages, and maintains homeostasis, for example, by altering how neurotrophic factors and N-methyl D-aspartate (NMDA) receptor subunits in the hippocampus are expressed (Bercik et al., 2011a; Heijtz et al., 2011). The main ways that the microbiota can influence the development and function of the nervous system are biological networks, including direct and indirect transmission via chemical transmitters, the immune system, neuronal pathways, and endocrine pathways, as shown in Figure 2.

### 3.1. Microbiota and neurotransmitters

Gut microbes can help regulate bodily functions and alter behavior in their animal host through chemical interactions with the nervous system, including both “direct” and “indirect” communication (Morais et al., 2021). Microorganisms have the ability to synthesize some of the neuroactive compounds themselves as well as stimulate the production of other metabolites and neurotransmitters by the host that regulate gut-brain signaling (Poutahidis et al., 2013). The microbiota is also required for the normal maturation, activation, and development of microglia, which are innate immune cells in the brain (Zheng et al., 2020). It seems that immune programming by microglia is regulated by signals from microbial metabolism because administering bacterial-derived short-chain fatty acids (SCFAs) to germ-free (GF) mice restores microglial shape and function (Erny et al., 2015). Microbial-derived molecules signaling to the brain. Neurotransmitters such as dopamine, serotonin, norepinephrine, glycine, and gamma-aminobutyric acids are produced by the intestinal microbiota, and each has a specific impact on brain γ-aminobutyric acid (GABA). Imbalances in these neurotransmitters can lead to disorders such as AD, PD, autism spectrum disorder, anxiety disorders, and depressive disorders (Chen et al., 2021).

For example, *Bifidobacterium infantis* increases blood plasma tryptophan levels, which affects central serotonin transmission; GABA can be produced by *Lactobacillus* and *Bifidobacterium*; noradrenaline can be produced by *Escherichia, Bacillus*, and *Saccharomyces* species; serotonin can be produced by *Streptococcus, Candida, Escherichia*, and *Enterococcus* species; dopamine can be produced by bacteria; and acetylcholine can be produced by *Lactobacillus* (Lyte, 2014a). SCFAs, a type of direct signaling, are lipids generated by intestinal microbes through the fermentation of dietary fiber that have the ability to influence the immune system, epigenetics, and neuroplasticity in the CNS (Dalile et al., 2019). The brain, energy balance, and metabolism are all impacted by SCFAs, which include butyrate, propionate, and acetate and are vital metabolic byproducts of gut microbial activity (Dinan et al., 2015). Additionally, SCFAs serve as endogenous ligands for orphan G protein–coupled receptors (GPCRs), and intracellular SCFAs regulate gene expression by preventing histone deacetylases. In addition, SCFAs interact with vagal afferents, which affects inflammation and hormone regulation. The interactions of SCFAs with particular cellular systems and gut-brain signaling pathways support the idea that SCFAs can play a significant role in GMB communication (Dalile et al., 2019). Through indirect chemical communication, the microbiota also affects the neurological system and behavior, as evidenced by the microbial regulation of the neuroendocrine system (Sudo et al., 2004). Gut microbiota can affect their host’s appetite and eating patterns by changing the endocrine signals produced by enteroendocrine cells (EECs) in the gut epithelium, which involves the production of the hormone glucagon-like peptide 1 (GLP1) (Aresti Sanz and El Aidy, 2019). The microbiota in the gut can produce neurotransmitters on their own and can also stimulate the creation of these chemicals in their animal hosts. For example, a number of microbes, including *Escherichia* spp., *Bacteroides, Bifidobacterium*, and its species, are known to generate the neurotransmitter GABA (Strandwitz et al., 2019). Bacteria have been demonstrated to be essential for the production of the neurotransmitter serotonin 5-hydroxytryptamine (5-HT) in animal mouse model systems (Clarke et al., 2013). Microbial metabolites such as indole, SCFAs, secondary bile acids, *α*-tocopherol, *p*-aminobenzoate, and tyramine have an impact on the generation and secretion of 5-HT by enteroendocrine cells (EECs) (Yano et al., 2015; Morris et al., 2017). Gut microbes synthesize SCFAs, 5-HT, dopamine, butyric acid, gamma amino acids, and gamma amino acids (Forsythe et al., 2014; Lyte, 2014b), and these substances are accessible between microbial cells (Forsythe et al., 2014). The gut, particularly intestinal cells, can synthesize large amounts of 5-HT, which affects the brain. Additionally, microbial enzymes can manufacture neurotoxins such D-lactic acid and ammonia (Manicassamy et al., 2010; Smith, 2015). These neuroactive metabolites, such as the neurotransmitters GABA, dopamine, noradrenaline and serotonin, amino acids (for example, tryptophan and tyramine) T lipopolysaccharide (LPS), short-chain fatty acids (SCFAs), long-chain fatty acids (LCFAs), trimethylamine-*N*-oxide (TAMO), and polysaccharide A (PSA), either directly or indirectly induce the migration of peripheral immune cells to the brain and are thought to cause neuroinflammation and influence CNS functions (Harms et al., 2018; Morais et al., 2021). Microbial-associated molecular patterns (MAMPs), which are released, also connect the CNS to the microbiota (Sampson and Mazmanian, 2015). MAMPs are molecules produced by gut microbes, such as double-stranded RNA, lipopolysaccharides, and lipoproteins, that are identified by a variety of receptors, especially Toll-like receptors (Akira and Hemmi, 2003; Schachtle and Rosshart, 2021). However, 5-HT and its metabolic precursor tryptophan concentrations in the hippocampus were decreased in germ-free mice, indicating a possible role for the gut microbiota in regulating 5-HT signaling pathways in the CNS (Clarke et al., 2013). In fact, it is difficult to evaluate how much microbial metabolism directly affects CNS activity, in part because we do not fully understand the average rate of transport for numerous microbial metabolites into the brain (Muller et al., 2020).

### 3.2. Endocrine pathway

SCFAs can alter the function of the gut-brain axis by regulating the production of gut hormones. The activation of G protein-coupled receptors (GPCRs) by SCFAs in the colon is the mechanism underlying the production of these gut hormones, which enhances the release of peptide YY (PYY) and glucagon-like peptide 1 (GLP1) from enteroendocrine L cells (Tolhurst et al., 2012; Psichas et al., 2015; Larraufie et al., 2018). These hormones in turn have the power to affect mood, memory, and learning. Through the use of free fatty acid receptors (FFARs), SCFAs can signal to the brain by directly activating vagal afferents (Dalile et al., 2019). GLP1 has many receptors throughout the body and can affect brain functions via both humoral and neuronal routes, including the CNS and PNS, as well as the heart, lungs, intestines and pancreas (Alvarez et al., 2005; Katsurada and Yada, 2016). GLP1 is involved in enhanced memory and learning in mice (Isacson et al., 2011), enhanced neuroplasticity and neuroprotection in the hippocampus (McClean et al., 2011; Porter et al., 2011), in animal models of AD, and in reduced βamyloid plaques and microglia activation (McClean et al., 2011). Another anorexic neuropeptide, PYY, reduces appetite and prevents gastric motility. In addition to the distal gastrointestinal tract’s L cells secreting it (colon and ileum), the hypothalamus and pituitary gland have the highest levels of PYY expression in the human brain, which is expressed throughout the brain (Morimoto et al., 2008). The most common form of circulating PYY is PYY_3–36_, a truncated form of the protein that preferentially interacts with the Y2 neuropeptide Y receptor (Murphy and Bloom, 2006). According to research conducted on animals, PYY affects both appetite and brain activity by either mechanisms that cross the blood–brain barrier (BBB) (Nonaka et al., 2003) or by activating vagal afferent pathways that connect to the gut wall’s lamina propria and myenteric plexus and transmitting to the brainstem (Koda et al., 2005; Waise et al., 2018). Other metabolic hormones that affect brain function and are influenced by SCFAs include ghrelin, leptin, and insulin; however, research on these hormones has been less focused than that on PYY and GLP1. Leptin is a hormone that induces weight loss that is mostly produced by adipose cells (Hube et al., 1996), and it is well known for regulating the body’s energy balance by activating its hypothalamic receptors to express orexigenic and anorexigenic neuropeptides such as neuropeptide Y and α melanoma-stimulating hormone, which reduces appetite (Elias et al., 1999).

### 3.3. Immune pathway

The immune system is influenced and directly affected by both the CNS and the gut microbiome. The gut microbiota has a significant impact on the development and function of the peripheral immune system (Zheng et al., 2020). The microbiota is necessary for the development and activation of innate immune cells in the brain (Abdel-Haq et al., 2019). The pathophysiology of psychiatric disorders may involve immune responses and inflammation (Miller and Raison, 2016). CNS-cytokine interactions affect brain functions and have an effect on neurocircuits that regulate motivation, motor activity, and mood (Capuron and Miller, 2011). Additionally, through the systemic immune system and circulating cytokines, the gut microbiota and the brain communicate (Hsiao et al., 2013). Immune cells directly penetrate the BBB and reach the CNS, or they can produce cytokines and chemokines in the brain (Morais et al., 2021). Cytokines are substances made in the intestine that can travel through the bloodstream and, under certain conditions, have an effect on the hypothalamus and other areas of the brain (El Aidy et al., 2014). The BBB is a physical barrier that separates the brain microenvironment from the rest of the body. It is made of tight junction proteins that connect the mural and microvascular endothelial cells (Morais et al., 2021). The BBB regulates the movement of molecules between the bloodstream and the cerebrospinal fluid of the CNS. Permeability of the BBB is influenced by the gut microbiota, as some reports show that GF mice have increased BBB permeability relative to control mice, partially due to reduced expression of tight-junction proteins such as occludin and claudin 5 (Braniste et al., 2014). The BBB allows it to effectively control the flow of chemicals, ions, and cells between the body’s environment and the brain (Engelhardt and Liebner, 2014). The BBB is important because it protects the brain against pathogens and unfavorable immune responses that could harm the neurons and the connections between them (Daneman and Prat, 2015). Many psychiatric disorders, such as major depression, schizophrenia, autism spectrum disorder, and obsessive–compulsive disorder, have been linked to microglial dysregulation (Frick et al., 2013). SCFAs have a direct impact on immune cells and immunological modulators to maintain homeostasis. The influence of SCFAs on intestinal mucosal immunity is well described by Corrêa-Oliveira et al. (2016). However, SCFAs may also have an impact on the peripheral immune system, modulating brain activity. By increasing the intestinal barrier and inhibiting the transfer of bacteria and bacterial metabolites or by direct contact between SCFAs and immune cells, which could decrease neuroinflammation in the brain, systemic inflammation may be reduced (Dalile et al., 2019). SCFAs regulate the maturation and activation of T lymphocytes, macrophages, dendritic cells (DCs), and neutrophils (Corrêa-Oliveira et al., 2016). Neutrophils, the most prevalent granulocyte type, are an essential part of the innate immune system and are produced in the bone marrow. They are the first to appear at the site of inflammation, and they exploit the production of cytokines to draw in other cells, such macrophages (Rodrigues et al., 2016). SCFAs have an immediate effect on neutrophils by regulating the production of proinflammatory cytokines such as tumor necrosis factor (TNF), possibly through histone deacetylase (HDAC) inhibition. By regulating the synthesis of chemokines such as CXC motif chemokine ligand 1 (CXCL1) and CXC motif ligand 8 (CXCL8), they also function as neutrophil chemoattractants. SCFAs affect neutrophil chemotaxis by causing free fatty acid receptor 2 (FFAR2) in these cells to become active (Rodrigues et al., 2016). SCFAs can affect adaptive immune responses by directly or indirectly affecting T-cell development and proliferation (Kim et al., 2014).

### 3.4. Neuronal pathways for gut–brain interactions

The gut and brain are physically linked through neurological connections. The most significant of these neural networks is the vagus nerve, which emerges from the brainstem and innervates the gastrointestinal tract and ENS (Yoo and Mazmanian, 2017). The most direct and well-studied link between the gut and the CNS, the vagus nerve, is another pathway through which gut microbes communicate with the brain (Fülling et al., 2019). Almost the whole digestive system is innervated by the vagus nerve, which has 80% afferent and 20% efferent fibers. The vagal afferent nerve terminals innervate multiple layers of the digestive wall, while the mucosal afferents end within the lamina propria of the intestinal mucosa (Waise et al., 2018). Vagal receptors sense inflammatory chemicals, dietary elements, bacterial metabolites, and regulatory gut peptides to transfer signals to the central nervous system (De Lartigue et al., 2011). However, there is some evidence that the bacteria in the gut can directly activate neurons. Toll-like receptors 3 and 7, which detect viral RNA, as well as Toll-like receptors 2 and 4, which detect peptidoglycan and lipopolysaccharide, are present in the enteric nervous systems of both mice and humans (Brun et al., 2013; Martin et al., 2018). *Bacteroides fragilis*, *Lactobacillus rhamnosus* (JB-1), and isolated polysaccharide A of *B. fragilis* have all been demonstrated to stimulate intestine afferent neurons ex vivo (Mao et al., 2013). Chronic treatment with *Bifidobacterium longum* NCC3001 reduced the symptoms of anxiety induced by persistent gut inflammation (Bercik et al., 2011b). The effects seen in these trials were eliminated when the vagus nerve’s integrity was compromised through vagotomy (Bravo et al., 2011; Bercik et al., 2011b). Additionally, microbial metabolites have the capacity to directly activate neurons. The receptors FXR and TGR5 are expressed in brain neurons, although healthy individuals have low or undetectable bile acid concentrations in these organs (Huang et al., 2016). Various studies have identified the superior cervical ganglion as the location of G protein-coupled receptor 41 (GPR41) and free fatty acid receptor 3 (FFAR3) receptors (Kimura et al., 2011), prevertebral ganglia (Won et al., 2013), submucosal and myenteric ganglia neurons (Nohr et al., 2013), sympathetic ganglia of the thoracic and lumbar sympathetic trunks, and vagal ganglion (Nohr et al., 2015), suggesting neuronal activation by microbially derived SCFAs. Neuronal innervation of the colonic epithelium is reduced in GF mice and restored by microbial colonization (De Vadder et al., 2018). Gut bacteria also aid in the development of enteric glial cells in mice, which are essential for maintaining neuronal networks and controlling gut homeostasis (Kabouridis et al., 2015; Aktar et al., 2020). The activity of enteric neurons can be influenced by the gut microbiota through chemical communication, according to a recent study showing that activating aryl hydrocarbon receptors in adult mice can affect gut motility by affecting the ENS (Obata et al., 2020).

## 4. Gut microbiota and neurological disorders

Neurological and neuropsychiatric disorders are associated with changes in the composition of the gut microbiota (Cryan et al., 2019; Tian et al., 2023). Neurological disorders are ailments of the central and peripheral nervous system that may harm the brain, spinal cord, cranial and peripheral nerves, autonomous nervous system, nerve roots, and neuromuscular plaque. Numerous conditions can lead to brain bleeding, including diseases of the blood vessels, disorders caused by issues with nervous system development, injuries to the spinal cord or brain, and brain tumors (Dugger and Dickson, 2017). A wide variety of neurological diseases are connected to dysbiosis of the human gut microbiome (Frank et al., 2007; Bibbo et al., 2017; Gavin et al., 2018; Kasselman et al., 2018; Duan et al., 2019). In contrast, patients with neurological diseases and healthy controls have dramatically different microbiota compositions (Sampson et al., 2016; Blacher et al., 2019; Valles-Colomer et al., 2019). Importantly, communication along the gut microbiota–brain axis occurs throughout life, as seen in diseases of neurodevelopment (for example, ASD), neurodegeneration (for example, PD and AD) and behavior (for example, depression and anxiety) (Figure 1). According to some recent studies in animals and humans, most of which were association studies, modifications in microbial diversity are linked to negative health outcomes and may cause alterations in the CNS (Table 1); these alterations are associated with ASD, depression, and anxiety (Felice and O'Mahony, 2017). Other studies have reported additional links between the microbiota composition and depression, anxiety, and ASD (Bercik et al., 2010; Sekirov et al., 2010; Claesson et al., 2012). Thus, the composition of the microbiota, which evolves over time, may have implications in brain function. In this Perspective, we review recent developments in the field of neuromicrobiology, particularly the links between the gut microbiota and neurological disease. In exploring the role that gut microbes play in neurological disorders, we specifically focused on ASD, AD, PD, depression, and anxiety disorders.

**Table 1:** Relationship between neurological disorders and gut microbiota.

Neurological disorders	Animal model	Causation vs. association	Changes in microbiota	Reference
Alzheimer’s disease	Human	The first evidence that the bacterial population, viral load, and progress of AD symptoms may be related. Each pathogen effects cognitive decline	Associated with infectious load, being viral (HSV-1 and CMV) or bacterial Helicobacter pylori Chlamydia pneumoniae, and Borrelia burgdorferi,	Bu et al. (2015)
Alzheimer’s disease	Human	Probiotic treatment did not meaningfully change other factors including oxidative stress and inflammation, but it may have a good impact on AD patients’ cognitive function.	probiotic supplementation containing: *Bifidobacterium bifidum, Lactobacillus casei, Lactobacillus fermentum*, and *Lactobacillus acidophilus*	Akbari et al. (2016)
Parkinson’s disease	Human	When mucosal and stool samples were analyzed with Parkinson’s disease were studied, several genes were shown to be downregulated in the stool microbiota of these people; the microbiota composition of the mucosal and stool samples was linked to substantial changes in patients with PD.	Bacterial increase: Proteobacteria, Betaproteobacteria, Coprococcus, Blautia, Akkermansia, Oscillospira, Roseburia, Bacteroides; bacterial decrease: Faecalibacterium, Firmicutes, class Clostridia	Keshavarzian et al. (2015)
Parkinson’s disease	Human	change in the fecal microbiota may contribute to the development of PD; Prevotellaceae was decreased in people with Parkinson’s disease, and a high abundance of this genus was not indicative of having PD; Prevotellaceae may serve as a biomarker to rule out PD because of their great abundance.	Bacteria decrease, Provotellaceae; the abundance of Ruminococcaceae could be associated with levels of Provotellaceae	Scheperjans et al. (2015)
Autism	Human	Autism symptoms and gastrointestinal (GI) alterations are related, and the development of autism may be influenced by an imbalance of bacteria associated with a healthy state.	Bacteria increase: *Lactobacillus*, Bacteria decrease: *Bifidobacterium* and *Enterococcus*; autism group more probable to have increased levels of *Bacillus* and reduced *Klebsiella oxytoca*	Adams et al. (2011)
Autism	Human	Children with autism have higher concentrations of *Suterella* spp. in their feces, and *Ruminococcus* torques is also more prevalent and may be linked to GI issues in these kids.	Bacteria increase: *Ruminococcus torques* and *Suterella*	Wang et al. (2013)
Autism	Human	A less diversified microbiome was found in autistic children, and the intestinal microbiota was linked to GI problems.	Bacteria reduction: *Veillonellaceae*, *Coprococcus*, and *Prevotella*; main phyla in microbiota of patients with autism: Bacteroidetes and Firmicutes; most rich genera: *Akkermansia*, *Bifidobacterium*, *Bacteroides*, *Faecalibacterium*, and *Subdoligranulum*	Kang et al. (2013)
Autism	Human	Detected a connection between bacterial populations and genes expressed in the colon of autistic children; the source of these intestinal abnormalities is still under investigation.	Bacteria increase; Bacteroidetes to Firmicutes ratio Lachnospiraceae and Ruminococcaceae, Betaproteobacteria, Bacteria decrease: Bacteroidetes	Williams et al. (2011)
Depression	Human	*Bifidobacterium* and *Lactobacillus* are less prevalent in people with major depressive disorder; fecal samples were examined to visualize the relationship between the bacterial population and irritable bowel syndrome (IBS).	Bacteria decrease: *Bifidobacterium* and *Lactobacillus*	Aizawa et al. (2016)
Depression	Rat	Probiotics normalized the immunological response, improved behavioral issues, and balanced noradrenaline levels in addition to reducing depressive symptoms.	Probiotic supplement comprising Bifidobacterium infantis 35,624	Desbonnet et al. (2010)
Depression	Mice	GF animals colonized with a “depression microbiota” had additional symptoms compared to control GF animals	Bacteria growth: Lactobacillaceae Coriobacterineae, Clostridiales, Streptococcaceae, Actinomycineae Lachnospiraceae Erysipelotrichaceae, Ruminococcaceae, and Eubacteriaceae: Bacteria reduction: Acidaminococcaceae, Rikenellaceae, Lachnospiraceae, Veillonellaceae, Bacteroidaceae, and Sutterellaceae	Zheng et al. (2016)
Depression		An improved understanding of the association between the microbiota and occurrence of particular bacteria with symptoms associated with depression resulted from examination of fecal samples from people with and without depression.	Bacteria increase: Rikenellaceae Enterobacteriaceae, Acidaminococcaceae, Porphyromonadaceae, and Fusobacteriaceae, Bacteria lessening: Prevotellaceae Erysipelotrichaceae, Lachnospiraceae., Veillonellaceae Bacteroidaceae, and Ruminococcaceae	Jiang et al. (2015)
Anxiety	Humans	Probiotic administration has been linked to better mental health; nevertheless, this probiotic combination had no negative effects on the hypothalamic–pituitary–adrenal (HPA) axis.	Probiotic supplement *Lactobacillus casei*, *Bifidobacterium logum*, *LA5* and *Bifidobacterium lactisBB12, Bifidobacterium breve*, *Lactobacillus acidophilus, Lactobacillus rhamnosus*, *Lactobacillus thermophilus, bulgaricus, Streptococcus*	Mohammadi et al. (2016)
Anxiety	Rats and humans	In rats, the probiotic complex was linked to decreased anxiety, and healthy humans showed improved psychological effects.	Probiotic supplement comprising *Lactobacillus helveticus R0052,* and *Bifidobacterium longumR0175*	Messaoudi et al. (2011)

### 4.1. Alzheimer’s disease

Alzheimer’s disease (AD) affects approximately 50 million people globally and is the most frequent cause of progressive chronic and irreversible neurological disease and the most common type of dementia in elderly individuals. As the condition progresses, symptoms that impair thinking and memory can seriously compromise even the most basic daily activities (Scheltens et al., 2016; Rutsch et al., 2020). Loss of neurons and progressively worsening synaptic dysfunction are symptoms of AD (Tiraboschi et al., 2004; Alzheimer's, 2016). AD is caused by the formation of aggregates of polymerized forms of β-amyloid precursor protein (Aβ) in soluble multimeric and/or insoluble amyloid deposits in the brain that trigger a cascade of pathological events leading to neurofibrillary tangles, aggregates of hyperphosphorylated tau proteins, formation of neurofibrillary lesions, and ultimately dementia (Scheltens et al., 2016). The inflammasome and its products have been connected to the pathogenesis of AD since a greater expression of IL-1β and IL-18 has been observed in the microglia, astrocytes, and neurons that surround Ab plaques or in the plasma of AD patients (Malaguarnera et al., 2006; Ojala et al., 2009). Peripheral blood mononuclear cells (PBMCs) from AD patients also showed greater expression of NLRP3, ASC, caspase-1, caspase-5, IL-1β, and IL-18 (Saresella et al., 2016). Patients with tauopathies, which are neurodegenerative diseases characterized by the accumulation of aberrant tau protein in the brain, typically exhibit increased levels of cleaved caspase-1 and ASC as well as mature IL-1β in the cortex (Ising et al., 2019). Important evidence links neuroinflammation caused by the NLRP3 inflammasome to the development and progression of AD. AD pathogenesis has been associated with a number of microbiological causes (Atarashi et al., 2011; Geuking et al., 2011). Compared to controls, AD patients’ stool samples showed higher levels of Bacteroidetes and lower levels of Firmicutes and Actinobacteria. Ruminococcaceae, Turicibacteraceae, and Clostridiaceae were all Firmicutes families where AD patients had lower abundances (Vogt et al., 2017). According to several studies, there may be mechanistic links between the pathophysiology of AD and other microbes, such spirochaetes, fungi, and Chlamydia pneumoniae (Lim et al., 2014; Stojkovi et al., 2020). In recent studies, the gut microbiota has also been connected to the etiology of AD. A metabolite microbiota-derived protein found in the cerebral fluid of AD patients and connected to two disease-related biomarkers (phosphorylated tau and phosphorylated tau/A-42) raises the possibility that the gut microbiome plays a role in the etiology of AD (Vogt et al., 2018). When comparing fecal microbiomes and fecal SCFAs between AD-affected mice and wild-type mice at various ages, dramatic increases in Proteobacteria and Verrucomicrobia and marked decreases in Butyricicoccus and Ruminococcus were observed in AD mice, indicating altered microbiota composition and diversity. The decreased level of SCFAs further indicates alterations in many metabolic pathways (Zhang et al., 2017). It was demonstrated that, compared to non-transgenic wild-type mice, the gut microbiota diversity of the commonly utilized APP/PS1 double transgenic mice—which produce a chimeric mouse/human amyloid precursor protein (APP) and a mutant human presenilin 1 (PS1)—was markedly changed. Additionally, compared to healthy control mice with gut microbiota, germ-free APP/PS1 transgenic animals show a striking reduction in the degree of cerebral *β*-amyloid pathology (Harach et al., 2017). Bäuerl et al. (2018) reported similar findings about the shift in microbiota composition in the transgenic APP/PS1 mouse model, which shows increased numbers of the closely related inflammatory Erysipelotrichaceae family. Furthermore, germ-free APP/PS1 mice showed decreased amyloid pathology compared to conventional mice (Radde et al., 2006).

### 4.2. Parkinson’s disease

Parkinson’s disease (PD), which affects more than 1% of the elderly population and 0.3% of the general population worldwide, is the second most prevalent neurodegenerative condition after AD (Tysnes and Storstein, 2017). PD is a progressive neurodegenerative disorder characterized by the inability to control voluntary movements as a result of severe alterations in the function of the substantia nigra and striatum. The degradation of dopaminergic neurons, the accumulation of phosphorylated versions of the neuronal protein α-synuclein (αSyn), mitochondrial malfunction, an excess of reactive oxygen species, and a rise in microglia activation are some of these alterations (Blandini et al., 2000). Inflammation and α-synuclein misfolding are both key pathological mechanisms underlying α-synucleinopathies such as PD (Lema Tom et al., 2013). The pathogenesis of PD largely depends on the accumulation of α-synuclein. The gene for α-synuclein has five exons and is located on chromosome 4q21.3-q22. -synuclein is a protein with 140 amino acids (Mehra et al., 2019). PD symptoms include tremors, trouble walking, a hunched posture, and muscle rigidity. Gastrointestinal issues, most frequently constipation, may affect up to 80% of patients with Parkinson’s disease (Chen et al., 2015) and can precede PD diagnoses by many years (Cersosimo et al., 2013). Growing evidence suggests that gut dysbiosis contributes to the onset, development, and progression of PD (Zhu et al., 2022). Comparing patients with prodromal and/or clinically diagnosed PD to subjects under control, we found that these patients had dysbiosis of the gut microbiota. The general organization and composition of the gut microbiota associated with PD have been examined using culture-independent high-throughput sequencing techniques, and features of the altered microbiota profiles in PD patients have been found (Zhu et al., 2022). Numerous earlier studies found that PD patients had higher α-diversity but lower bacterial diversity than healthy people (Qian et al., 2018; Barichella et al., 2019). Additionally, one study revealed that there were differences in *β*-diversity (between samples) between PD patients and controls (Boertien et al., 2019). There has been a connection between the clinical characteristics of PD and the decline in bacterial diversity, which is primarily assessed using α-diversity indexes such as Shannon and Simpson. According to a recent study by Heinzel et al. (2021), certain symptoms of PD may be particularly related to the prodromal microbiome, including constipation, possible rapid eye movement sleep behavior disorder (RBD), physical inactivity, smoking, urate levels, and subthreshold parkinsonism. Contrary to sex, inactivity, suspected RBD, constipation, and smoking, which were all connected to *β*-diversity, were constipation, occupational solvent exposure, and the three previously mentioned variables. Age and medications that reduce urate were linked to both *α* and *β*-diversity (Heinzel et al., 2021). However, research by Plassais et al. (2021) revealed that the gut microbiome’s α-diversity is not a biomarker of PD. The intestinal permeability and inflammation caused by the gut dysbiosis associated with PD, such as increased Akkermansia and decreased SCFA-producing bacteria, can facilitate the exposure of the intestinal neural plexus to toxins such as lipopolysaccharide (LPS) and pesticides, which can cause abnormal α-synuclein fibril aggregation and the development of Lewy bodies (Hirayama and Ohno, 2021). Despite people with other diseases, people with PD have a different microbiome composition from people who are healthy or have other neurological disorders (Hasegawa et al., 2015; Keshavarzian et al., 2015; Scheperjans et al., 2015). The intestinal flora in PD patients is lacking in bacteria that produce SCFAs (mostly butyrate), such as taxa from the Lachnospiraceae family (Hill-Burns et al., 2017; Petrov et al., 2017; Barichella et al., 2019) and *Faecalibacterium prausnitzii* (Keshavarzian et al., 2015; Unger et al., 2016), which have known anti-inflammatory properties. Additionally, certain bacterial species, such as Proteus mirabilis, which causes mice to develop motor impairments, may be the cause of PD-like disease (Choi et al., 2018). Prospective long-term longitudinal microbiome investigations are required to track the development of the disease and characterize changes in the microbiome’s taxonomic composition that contributed to or may potentially have defined the disease state. Uncertainty persists regarding the precise way in which the gut microbiome may affect PD-related symptoms.

### 4.3. Multiple sclerosis

Multiple sclerosis (MS) is a neurological and inflammatory condition that affects over two million individuals worldwide. The main symptoms of this condition include demyelination, axonal loss, lymphocyte infiltration into the CNS, and neuroinflammation. Some of the clinical signs of MS include ataxia, poor coordination, hyperreflexia, stiffness, visual and sensory impairment, fatigue, and cognitive deficits. The majority of patients suffer a kind of disease known as relapsing–remitting, which is characterized by a gradual but significant deterioration in neurological function and a progressive reappearance of symptoms (McFarland and Martin, 2007). Most patients have brain lesions or lesions in the brain and spinal cord; however, some people only have lesions in the spinal cord (McFarland and Martin, 2007; Rutsch et al., 2020). Microbes (and the substances they secrete or toxins they produce) are a significant contributor to the pathophysiology of MS among environmental variables (Ronchi et al., 2016; Rutsch et al., 2020). MS patients have a different microbiome composition than healthy individuals (Oleskin and Shenderov, 2016). It is interesting to note that even MS patients with active disease have a different microbiome from those who are in remission, whose microbiota is more comparable to that of healthy controls (Bhargava and Mowry, 2014; Chen et al., 2016; Jangi et al., 2016; Pröbstel and Baranzini, 2018). Greater Firmicutes abundance and the absence of Fusobacteria in pediatric MS patients were associated with a shorter time to relapse (Tremlett et al., 2017). Compared to healthy people, fecal samples from people with MS show alterations in the richness of *Mycoplana, Dorea, Pseudomonas, Blautia,* and *Akkermansia* species (Mangalam et al., 2017). Attenuated multiple sclerosis-like disease appears in preclinical models in GF mice (Lee et al., 2011), and mice receiving the intestinal microbiota of MS patients experienced more severe experimental autoimmune encephalomyelitis and had lower proportions of anti-inflammatory regulatory T cells than mice receiving the microbiome of healthy individuals (Berer et al., 2017; Cekanaviciute et al., 2017). The remarkable finding was that transplanting the intestinal microbes of the MS twins into GF animals, which are genetically predisposed to developing experimental autoimmune encephalomyelitis (EAE), was sufficient to promote the illness in vivo with a significantly higher incidence than transplanting the microbes of the healthy twins (Berer et al., 2017). Interestingly, immune cells from mice that received MS-derived samples produced less IL-10 than cells from animals that had their microbiota from healthy twins colonize (Berer et al., 2017). In mice inoculated with healthy fecal samples, the neutralization of IL-10, one of the main regulatory cytokines, increased the incidence of disease (Berer et al., 2017). This important finding demonstrated how the human microbiome may produce particular immune system changes that may be the cause or consequence of the onset of MS. Uncertainty persists regarding whether this role plays a crucial part in the beginning and development of the disease. In light of this, there is considerable interest in the variations in the microbiota of MS patients compared to healthy controls.

### 4.4. Autism spectrum disorder

Autism spectrum disorder (ASDs) are a set of neurological development changes marked by difficulties with social interaction and communication as well as stereotyped and repetitive conduct (Maiuolo et al., 2021). Constipation, diarrhea, abdominal pain, flatulence, and intestinal gas are common among people with ASD problems (23–70%) and are frequently comorbid with gastrointestinal diseases (Mulle et al., 2013). The gut microbiota mediates the levels of chemical transmitters such as GABA, glutamate, oxytocin and serotonin 5-HT complex in ASD. Due to the low-grade inflammation that ASD patients experience, microbial influences on the immune system may also be very important in determining neuroimmune responses in ASD. New technologies are being applied in this rapidly expanding field of research as it becomes obvious how much microbial metabolites, including taurine, bile acid metabolites, SCFAs, and 5-aminovaleric acid, affect ASD symptoms (Morais et al., 2021). There are few and generally inconsistent ASD studies that highlight the role of the microbiome in pathogenesis. However, there are a few that highlight the differences in bacteria such as Firmicutes, Clostridiales, Prevotella, Bifidobacterium, and Clostridium perfringens, species that are seen between ASD patients and controls (Ho et al., 2020). This results in a change in the composition of the gut microbiota, a reduction in dietary quality, and a deficiency in nutrients (Berding and Donovan, 2016). The scientific literature data generally show a reduction of Bacteroides with a ratio (% ASD child/% control children) equal to 0.71; a reduction of Bifidobacterium with a ratio (% ASD child/% control children) equal to 0.52; a reduction of Escherichia coli with a ratio (%) equal to 0.3; an increase in Faecalibacterium with a ratio (%) equal to 1.32; and an increase in Lactobacillus Clostridium is still present in a largely unchanged amount (Tomova et al., 2015). It is evident that these neurological disorders are accompanied by reduced amounts of beneficial bacteria and larger levels of deadly bacteria, even though it cannot be said that certain bacteria are compatible and connected with the start of ASD (Iglesias-Vázquez et al., 2020). The gut microbiota and its metabolites may be crucially significant in the pathophysiology of ASD (Xu et al., 2019).

### 4.5. Anxiety and depression

Anxiety and depression are mental and neurological disorders that affect 25% of the global population. These two pathological conditions appear to be intimately related: in fact, 85% of people with depression and 90% of people with anxiety disorders both experience considerable anxiety (Bui and Fava, 2017; Maiuolo et al., 2021). Early and late stages of these pathologies have significantly different clinical signs (Groeneweg-Koolhoven et al., 2017). Teenage suicide deaths have increased in recent decades as a result of the rise in depressed symptoms (Jorm et al., 2017; Matsumoto et al., 2017; Weinberger et al., 2018; Twenge et al., 2019). The relationship between anxiety and depression and changes in the stability and composition of the gut microbiota has been thoroughly investigated (Tognini, 2016; Zhao et al., 2018; Bastiaanssen et al., 2019). Numerous studies have recently focused on the relationship between the intestinal microbiota and people who suffer from anxiety and mood disorders. In particular, evidence from human research has demonstrated that when taking into account microbial diversity and taxonomic compositions, there is frequently some variation in the fecal microbiota between patients and healthy controls. Additionally, it was revealed that certain bacteria were linked to clinical traits and metabolic or inflammatory profiles (Huang et al., 2019). There have been some studies on human microbial diversity, but the majority of them have been unable to show a connection between low microbial diversity and depressive disorders (Chen et al., 2014; Naseribafrouei et al., 2014; Zheng et al., 2016). Despite the fact that only one study found that individuals with major depressive disorder (MDD) had a higher alpha diversity of the gut microbiota than healthy subjects, alpha diversity is the number of species that can be detected in a microbial ecosystem (Jiang et al., 2015). Comparing patients with MDD to drug-responders with healthy controls, patients with MDD showed higher fecal a-diversity (higher levels of Enterobacteriaceae and Alistipes but lower levels of Faecalibacterium). Because of this, the authors reported a link between Faecalibacterium and the intensity of depression symptoms that was negative (Jiang et al., 2015). Interesting changes in the fecal microbiota have also been found in patients with anxiety disorders. They observed that patients with generalized anxiety disorder (GAD) had lower levels of microbial diversity and richness, which was correlated with lower levels of short-chain fatty acid producers such as *Eubacterium rectale* and *Fecalibacterium* and higher levels of *Ruminococcus, Escherichia, Shigella,* and *Fusobacterium* (Jiang et al., 2018). According to another study, probiotics (*Bifidobacterium bifidum, Lactobacillus acidophilus,* and *Lactobacillus casei*) administered to MDD patients dramatically reduced depression symptoms when compared to a placebo (Akkasheh et al., 2016). The potential of bacteria to produce 3,4-dihydroxyphenylacetic acid, a metabolite of dopamine, correlates favorably with mental health according to fecal metagenomic data, which raises the possibility that microbes play a role in the production of different neuroactive molecules during depression than under normal conditions (Valles-Colomer et al., 2019). *Lactobacillus rhamnosus* releases GABA and activates GABA receptors in the brain (that is, GABA _Aα2_ and GABA _B1b_ receptors) and has been revealed to attenuate depression and anxiety-like behaviors in mice (Bravo et al., 2011).

### 4.6. Stroke

Stroke is the second leading cause of death worldwide. The morbidity and mortality of stroke grow in many countries, contributing to financial burden and loss of life quality and thus diminishing the national happiness index. Approximately 15 million people around the world are victims of a stroke every year (Feigin et al., 2017). They may occur due to modifications in various diseases, such as cerebrovascular disease, atherosclerosis, dyslipidemia, diabetes, and arterial hypertension (Goldman et al., 2022). However, to date, few studies have focused on exploring the correlation between hemorrhagic stroke and the gut microbiota. GM microflora may be involved in the development of stroke and/or brain injuries (Singh et al., 2016). Studies have reported that ischemic stroke accounts for ~80% of all strokes (Sadler et al., 2020), and the gut microbiota plays an essential role in the pathogenesis and prognosis of ischemic stroke. Multiple studies have shown that ischemic stroke significantly changes the gut microbiota composition (Ling et al., 2020; Xiang et al., 2020; Xu et al., 2021). Patients suffering from transient ischemic attack or stroke have been found to have opportunistic pathogens such as *Desulfovibrio*, *Enterobacter*, *Megasphaera*, and *Osicillibacter*, as well as fewer beneficial or commensal pathogens such as *Bacteroides*, *Fecalibacterium*, and *Prevotella* (Yin et al., 2015). The abundance of Peptococcaceae and Prevotellaceae is linked to stroke severity (Tiwari et al., 2023). Recently, a preclinical study also suggested that alterations in the gut microbiota were associated with hemorrhagic transformation (HT). The relative abundance of *Proteobacteria* and *Actinobacteria* was significantly increased in HT rats after experimental stroke, indicating that the gut microbiota is involved in the progression of ischemic stroke (Huang et al., 2022). The precise role and mechanism of GM in the onset and progression of stroke and brain injury remain unknown. Although animal models have yielded fascinating results, more clinical research is needed to fully elucidate the potential of such microbial therapeutic modalities.

## 5. Conclusion and future directions

The gut microbiome is important for the host’s health and disease states, and most of the research on this subject to date has only revealed associations between certain clinical disorders and bacterial profiles. The gut microbiota has a substantial impact on both the physiology and pathophysiology of the brain due to the interaction between the intestine and neurological system in both directions. This communication takes place via a variety of pathways and involves the vagal nerve, neuroendocrine systems, neurotransmitters of the CNS, and inflammatory substances. The discussed evidence is accumulated from preclinical and clinical studies on gut microbiota, its dysbiosis and association with the development and progression of neurological disorder neurodevelopmental abnormalities to depression and Parkinson’s diseases, even if determining their exact mode of action requires more research, and probiotic supplement therapies are useful with promising therapeutic possibilities for neurological diseases. Probiotic supplement therapies are effective tools with considerable therapeutic potential for neurological disorders, even though determining their precise mode of action requires more research. Future studies in this field may provide insight into the connection between the microbiota and the CNS and developments in the treatment of neurological disorders. The fields of microbiology and neuroscience, as well as other disciplines, must proceed to work together to develop thorough and pertinent methods to ascertain mechanisms of action for outcomes that are currently observational, along with responsible efforts in translating these discoveries to improve human health. World’s major populations are suffering from neurological disorders, which are expected to rise by 13% by 2030. Hence, there is an urgency to develop more reliable biomarkers and feasible therapeutic options in view of the diseases’ pathogenicity. Multiple studies have shown that the GM is critical for brain development and function. In a number of preclinical and clinical research studies, the GIT microbiome in the GBA has been reviewed for its association with multiple neurological disorders, such as AD, MS, PD, ASD, epilepsy, stroke, and brain injury. However, deeper research is needed to understand the mechanism of action and function of GM in disease pathogenesis and its further applicability for therapeutic or prognostic purposes. However, the impact on the GM and the composition of their beneficial species in the GBA still need to be elucidated in future studies. Because many patients are given multiple medications, more research is needed to clarify any potential GM–drug interactions. The GM is a new line that separates human health from a variety of disorders, and future neurotherapeutic research will provide critical information on this topic. Despite recent developments in our understanding of the GBA, further research is required to determine whether this knowledge can be helpful in a clinical environment. Future studies must clarify the underlying links between the GM and various neurological diseases and determine whether treating the microbiota is a safe and effective course of treatment. It may be possible to develop techniques that target the gut microbiota to offer innovative, safe, and efficient therapy options for neurodegenerative disorders if traditional brain disorders are viewed comprehensively and now as entire conditions with a significant role for the gastrointestinal tract.

## Author contributions

HU and SA: original draft preparation and conceptualization. YT, C-qL, YC, LQ, MK, and IH: methodology. HU: review and editing of the manuscript. KL: supervision. All authors have read and agreed to the published version of the manuscript.

## Conflict of interest

The authors declare that the research was conducted in the absence of any commercial or financial relationships that could be construed as a potential conflict of interest.

## Publisher’s note

All claims expressed in this article are solely those of the authors and do not necessarily represent those of their affiliated organizations, or those of the publisher, the editors and the reviewers. Any product that may be evaluated in this article, or claim that may be made by its manufacturer, is not guaranteed or endorsed by the publisher.

## Abbreviations

GM: gut microbiota
GBA: gut-brain axis
CNS: central nervous system
AD: Alzheimer’s disease
PD: Parkinson’s disease
GI: digestive tract
MS: multiple sclerosis
ASD: autism spectrum disorder
ENS: enteric nervous system
GBA: gut-brain axis
HPA: hypothalamic–pituitary–adrenal axis
NMDA: *N*-methyl-d-aspartate
GABA: gamma-aminobutyric acid
EECs: enteroendocrine cells
GLP1: glucagon-like peptide 1
5-HT: 5-hydroxytryptamine
LPS: T lipopolysaccharide
LCFAs: long-chain fatty acids
TAMO: trimethylamine-*N*-oxide
PSA: polysaccharide A
MAMPs: microbial-associated molecular patterns
GPCRs: G protein-coupled receptors
PYY: peptide YY
GLP1: glucagon-like peptide 1
FFARs: free fatty acid receptors
BBB: blood–brain barrier
TNF: tumor necrosis factor
DCs: dendritic cells
HDAC: histone deacetylase
CXCL1: CXC motif chemokine ligand 1
CXCL8: CXC motif ligand 8
FFAR2: free fatty acid receptor 2
GPR41: G protein-coupled receptor 41
FFAR3: free fatty acid receptor 3
PBMCs: peripheral blood mononuclear cells
APP: amyloid precursor protein
PS1: presenilin 1
αSyn: α-synuclein
GAD: generalized anxiety disorder
EAE: experimental autoimmune encephalomyelitis
